# Femoral Nail and Cement Static Spacer Technique for the Treatment of Chronic Periprosthetic Knee Infection

**DOI:** 10.1016/j.artd.2025.101630

**Published:** 2025-02-15

**Authors:** Katherine Mistretta, Caroline Granger, Joseph Kromka, Andrew M. Schneider

**Affiliations:** Department of Orthopaedic Surgery, Washington University in St. Louis, St. Louis, MO, USA

**Keywords:** Periprosthetic joint infection, Static antibiotic spacer, Revision total knee arthroplasty, 2-stage

## Abstract

A 2-stage protocol is standard of care treatment in the United States for chronic periprosthetic joint infection of the knee. While many patients benefit from insertion of an articulating spacer, there are instances in which this is not feasible, and a static spacer is indicated. However, many static spacer techniques risk instability and lack durability. The ideal static spacer construct should provide immediate brace-free weight-bearing to maximize function during the spacer stage and, if needed, permit delayed reimplantation in the case of medically high-risk patients. Here, we describe our surgical technique for a femoral nail and cement static spacer in the treatment of chronic knee periprosthetic joint infection, a reproducible, stable, and durable construct essential to the armamentarium of the arthroplasty surgeon.

## Introduction

Periprosthetic joint infection after primary total knee arthroplasty (TKA) is a disabling and life-altering condition with an incidence of 1%-4% [[1], [2], [3], [4], [5], [6]]. Currently, the standard of care treatment in the United States consists of a 2-stage protocol, with the first stage including removal of the infected arthroplasty components and insertion of an antibiotic cement-loaded spacer [[13], [10], [11]].

Historically, spacers consisted of a block of antibiotic-loaded cement in the knee joint, which led to unacceptable rates of instability, surrounding soft tissue and bone destruction, and lack of function [10]. Today, spacer constructs may be articulating or static, with the best available literature suggesting no difference in infection eradication rates between articulating and static spacers. However, articulating spacers have been shown to provide a more pliable soft tissue envelope upon replantation, and a better ultimate knee range of motion [11,12].

Despite the functional advantages of contemporary articulating spacers, there remain circumstances in which an articulating spacer is not feasible and a static spacer is preferred. These include the presence of significant bone and soft tissue defects, collateral ligament insufficiency, and extensor mechanism disruptions [[10], [11], [12], [13]]. In these instances, the ideal static spacer would allow for ample antibiotic-cement volume in the joint space and metaphysis, provide immediate and full brace-free weight-bearing to maximize function, maintain limb length, and afford durability in the case of prolonged spacer retention [14]. Recently, static spacers employing intramedullary fixation have been described for increased inherent stability, using external fixator bars, tibial nails, Steinmann pins, fusion nails, or femoral nails [[15], [16], [17]]. Of these options, femoral nails are the most cost-effective, readily available, and largest diameter device without a central connection which suggests increased potential for full brace-free postoperative weight-bearing without implant failure.

Here, we describe our preferred femoral nail and cement static spacer technique for chronic knee periprosthetic joint infection. This technique is reproducible and reliable, and the construct may act as a functional antibiotic knee arthrodesis in situations of delayed reimplantation. In the patient case presented, static spacer was selected in favor of articulating spacer due to bone loss (flexion gap substantially larger than extension gap).

## Surgical technique

### Spacer insertion

**Figure 1:**
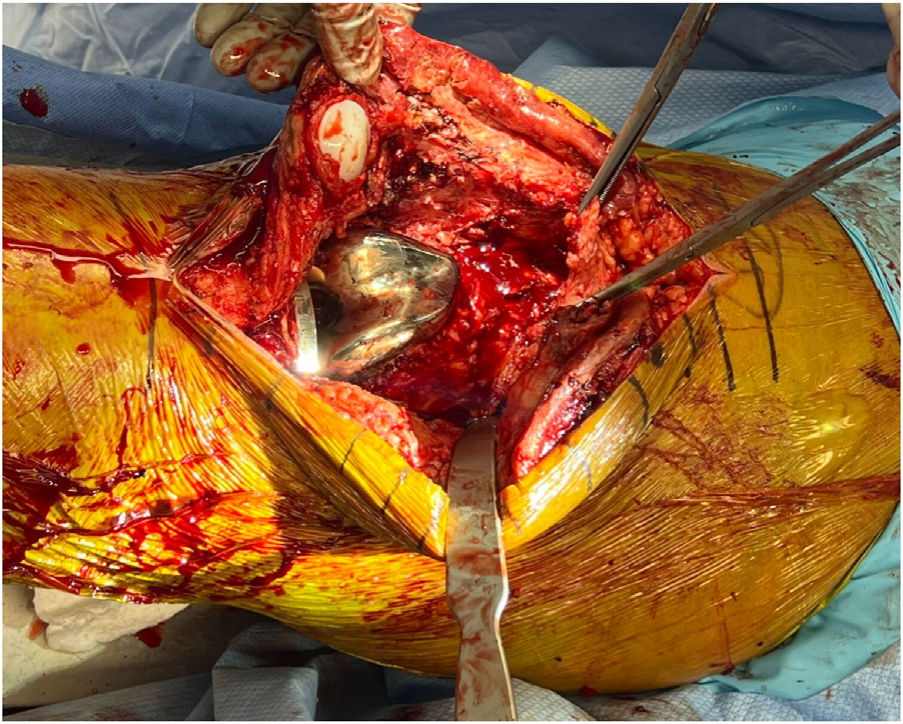
Full-thickness subcutaneous flaps should be elevated medially and laterally to define capsular layer. Medial parapatellar arthrotomy is created followed by radical soft tissue debridement of the medial and lateral gutters prior to removal of femoral, tibial, and patellar components.

**Figure 2:**
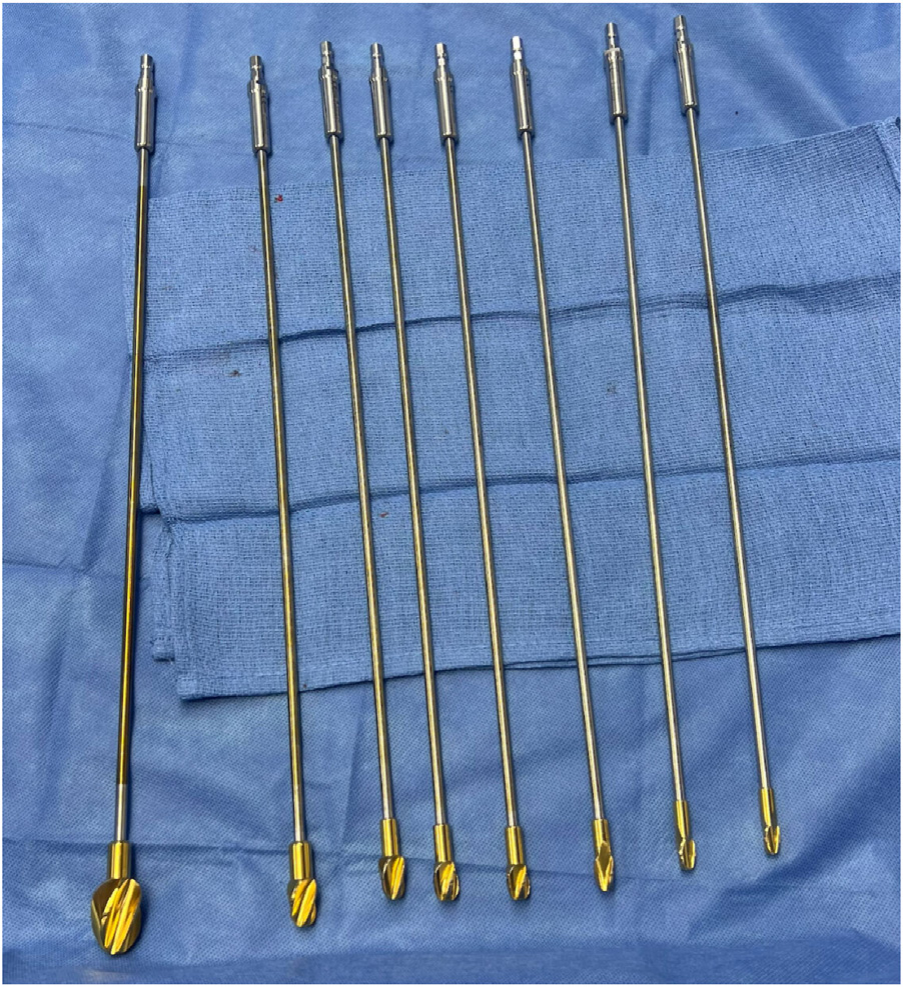
Sequentially larger reamers used for femoral and tibial preparation. The largest reamer (on the left) is of a 22-mm diameter and is used to create a cone-shaped bony defect in the tibial and femoral metaphyses to allow for cement to fill this region and obtain improved rotational control of the spacer.

Following sterile preparation and draping of the patient-positioned supine with nonsterile tourniquet high on the operative thigh, standard approach to the affected knee should be performed via previous midline incision. It is recommended that the foot be prepped into the field with the location of the dorsalis pedis pulse marked with a sterile marking pen to allow for intraoperative and postoperative pulse checks. We choose to double-drape the operative limb; the first set of drapes is removed following completion of debridement and a new set of clean instruments is used following removal of the “dirty” top drape. Full-thickness subcutaneous flaps should be elevated medially and laterally to define capsular layer and allow for adequate mobilization of soft tissues (Fig. 1). Medial parapatellar arthrotomy should be created followed by radical soft tissue debridement of the joint with procurement of at least 5 tissue cultures. All implants should be removed using the surgeon’s preferred technique, minimizing bone loss. Passage of flexible reamers into the femoral and tibial canal is recommended to remove any residual biofilm (Fig. 2).

The femur and tibia should be sequentially reamed using flexible reamers until cortical chatter is achieved. This step can be performed over a guidewire if the surgeon chooses (although not mandatory), and the goal for reaming should be up to size 14.5-15 mm as most femoral nails have a proximal diameter of about 13 mm. One should anticipate the need to ream for stems during subsequent surgery; therefore, only enough cortical bone should be removed to debride the canal and pass the nail. A large (22-24 mm) reamer should be used to create a cone-shaped bone defect in the tibial and femoral metaphysis to allow for cement to fill this region, thereby improving rotational control of the spacer construct. The nail will not be coated with cement proximally or distally, so these metaphyseal “plugs” will act as barriers between the effective joint space and the tibial and femoral canals. Distal femoral and proximal tibial bone ends may then be freshened using standard technique, creating cut surfaces perpendicular to the mechanical axis of the limb.

**Figure 3:**
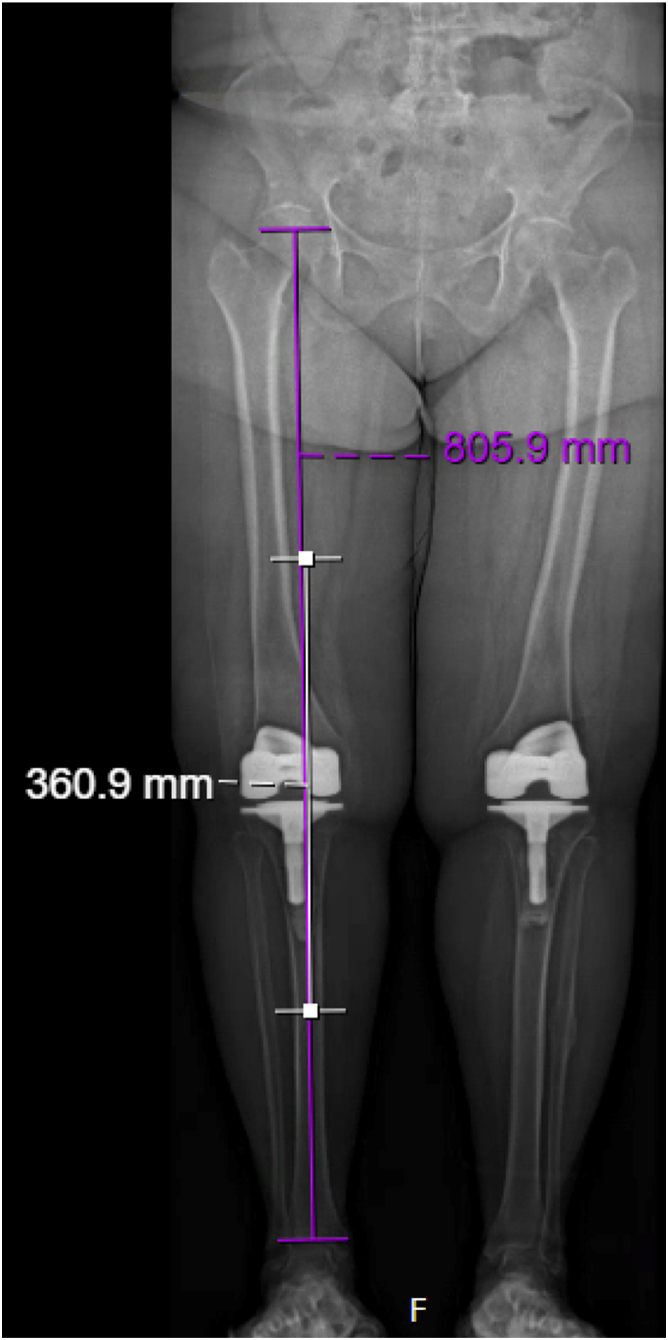
The length of the femoral nail is selected based on preoperative long length radiograph to engage both the tibial and femoral diaphyses, measured here as a 360-mm nail.

**Figure 4:**
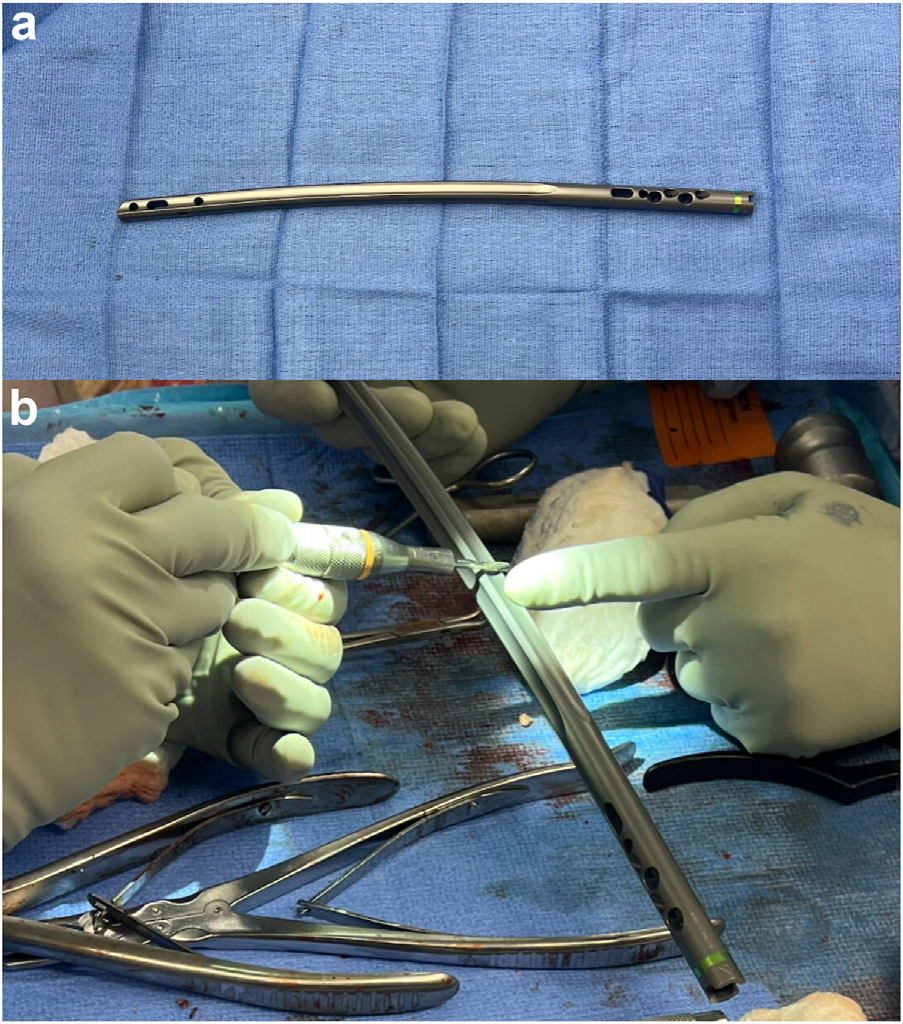
(a)Piriformis entry nail used demonstrates anterior bow built into the nail design which must be accounted for during insertion. (b) Utilization of a metal cutting burr to mark the center point of the nail on the side of the anterior bow of the nail for future use as reference following insertion into the femoral and tibial canal.

**Figure 5:**
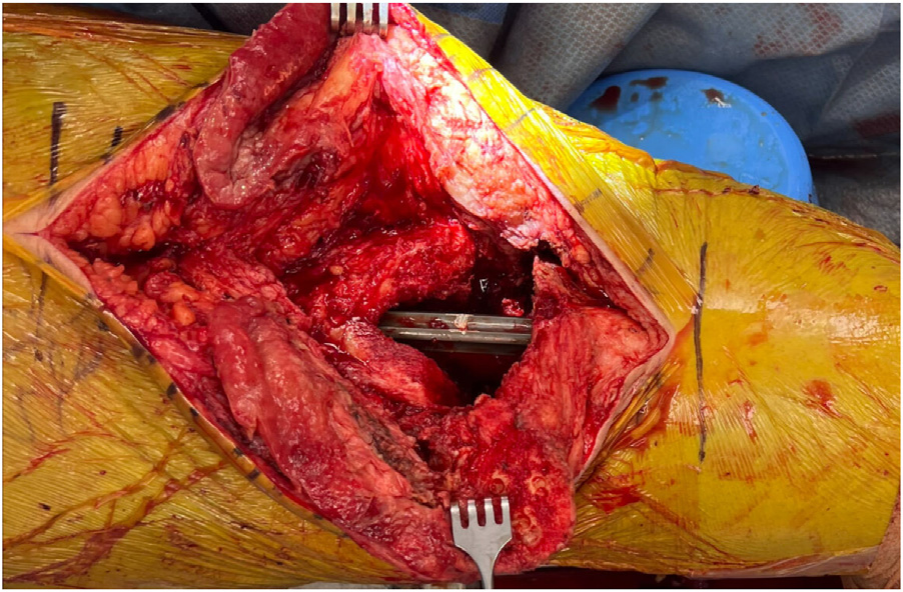
Trial insertion of the femoral nail performed prior to mixing cement, ensuring the mark on the anterior bow of the nail is facing anteriorly and is in the center of the joint.

The length of femoral nail should be selected based on preoperative calibrated X-ray or computed tomography scan (Fig. 3). Engagement of both tibial and femoral diaphysis should be planned when selecting nail length, which will normally fall within the 360-400 mm range. The tibial isthmus will normally be the limiting factor, as this is typically the narrowest point. Approximately 75-100 mm of the length of the nail engaged in the diaphysis of each bone is a reasonable goal. A piriformis-entry antegrade femoral nail is recommended (Fig. 4a). After the nail is selected, a metal-cutting burr should be used to mark the center point of the anterior bow of the nail for reference following insertion (Fig. 4b). Trial insertion of the femoral nail should be performed prior to mixing cement; insert the proximal end of the nail into the femur, reduce the joint, and then use a vice grip or pliers to slide the distal end of the nail down the tibia until the center mark is located at the center of the knee joint, ensuring that mark on the anterior bow is pointing anterior (Fig. 5). If there are difficulties passing the nail, the cause is usually mismatch of the bow of the nail to the native femur. In this case, the nail should be removed, and flexible reaming may be continued sequentially by half sizes until the nail can be successfully passed.

**Figure 6:**
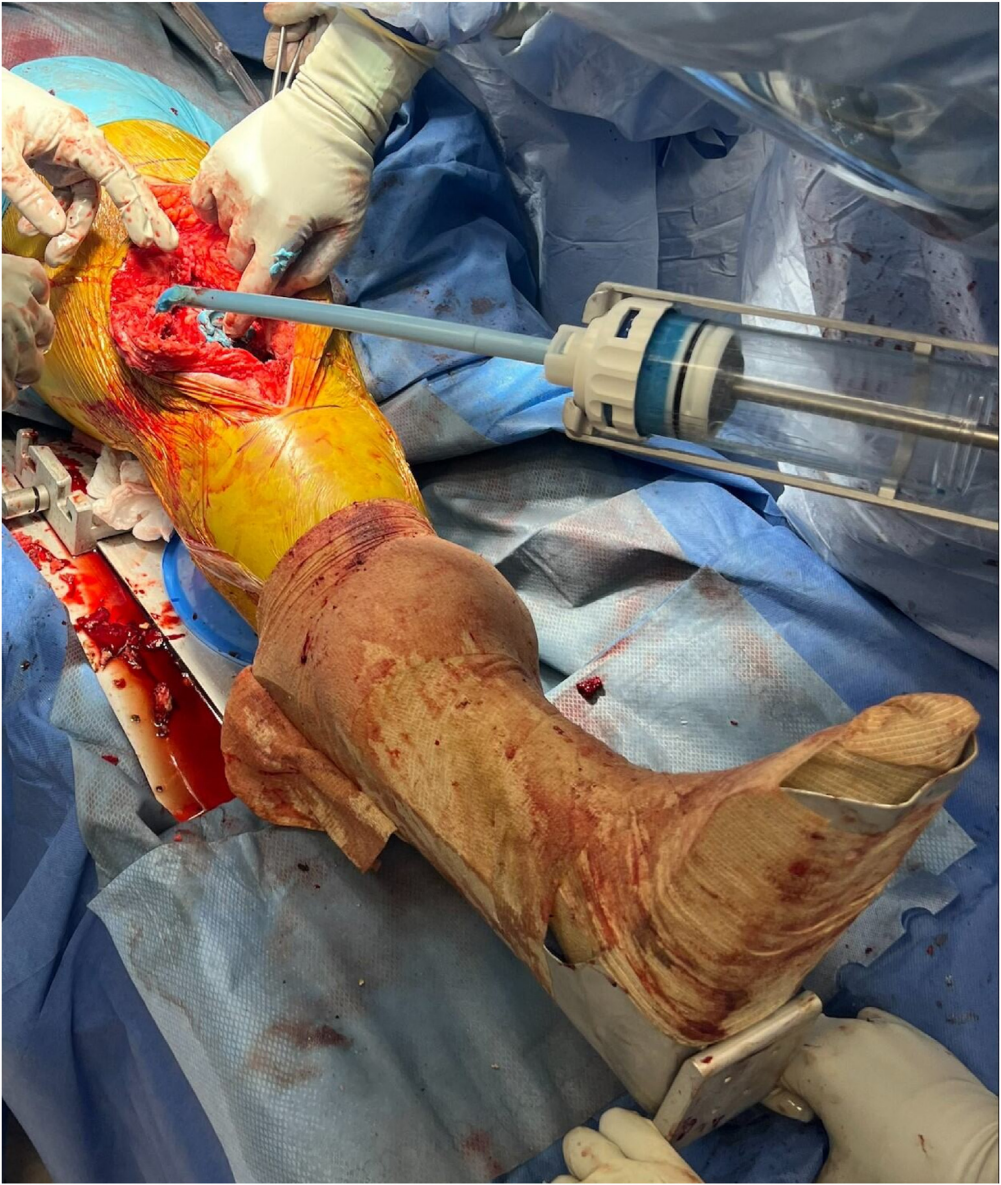
With the femoral nail in the appropriate position and orientation, cement is injected into the femoral and tibial metaphysis using a long cement gun tip while gentle traction is pulled by the surgical assistant.

**Figure 7:**
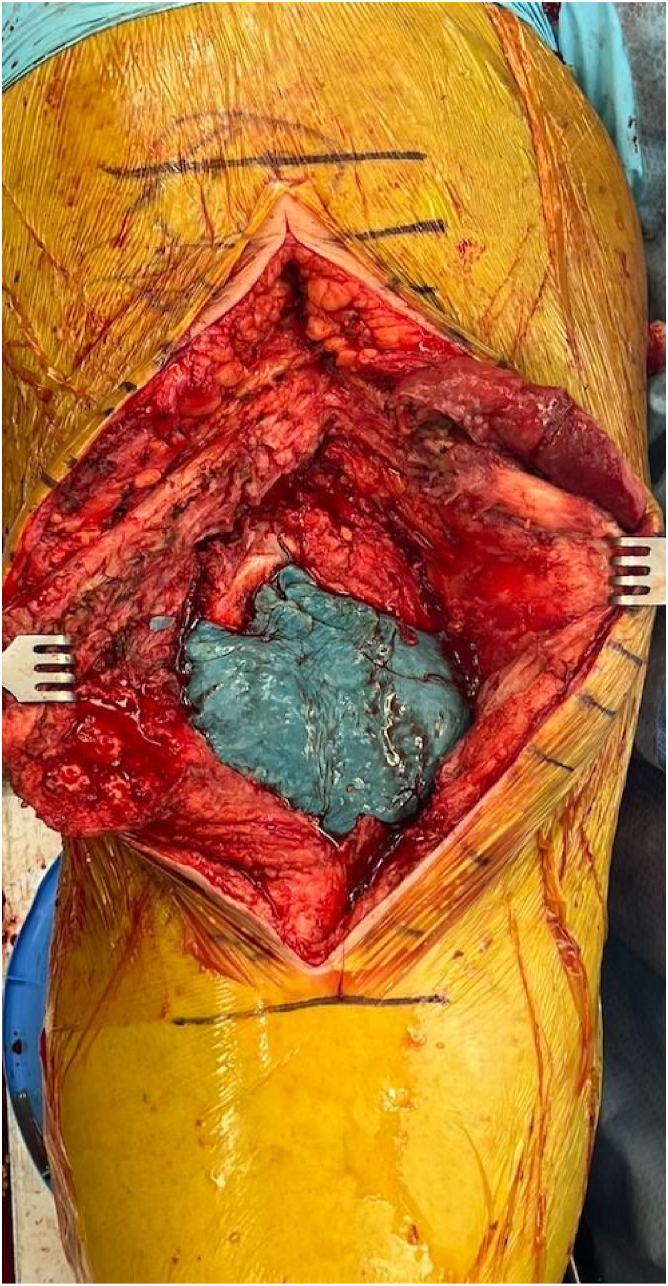
Final spacer construct prior to closure. Joint space is filled with as much cement as possible while allowing for arthrotomy closure.

At the time of implantation of the final construct, the tourniquet on the operative extremity should be inflated and the knee should be slightly flexed to about 5°-10° over a small towel bump. Appropriate hemostasis should be achieved prior to tourniquet inflation. Two mixing bowls are then simultaneously used to make antibiotic cement. Each mixing bowl contains 2 bags of antibiotic cement with 2 grams of vancomycin and 2.4 grams of tobramycin per bag to achieve appropriate viscosity. Methylene blue is added to each bowl when mixing for ease of future cement extraction. A cement gun with long nozzle and breakaway tip is then loaded with the cement mixture. With the femoral nail in place in the correct position and orientation, cement should then be injected into the femoral and tibial metaphysis using a long cement gun tip; the long tip can then be broken away and shorter tip used to completely fill the joint space (Fig. 6). While injecting the cement, gentle traction should be pulled by surgical assistant to allow for distention of the joint capsule and pericapsular ligaments, which will later be critical in achieving soft tissue closure during second-stage revision. Placing instruments to restrict posterior extrusion of cement within the joint space is up to surgeon discretion; an option for this would be 1-2 bent malleable retractors, but we have not found this to be necessary. The joint space should be filled with as much cement as possible while still allowing for closure of the arthrotomy (Fig. 7). This should be titrated while the cement is hardening, adding or removing cement from the joint space as needed.

**Figure 8:**
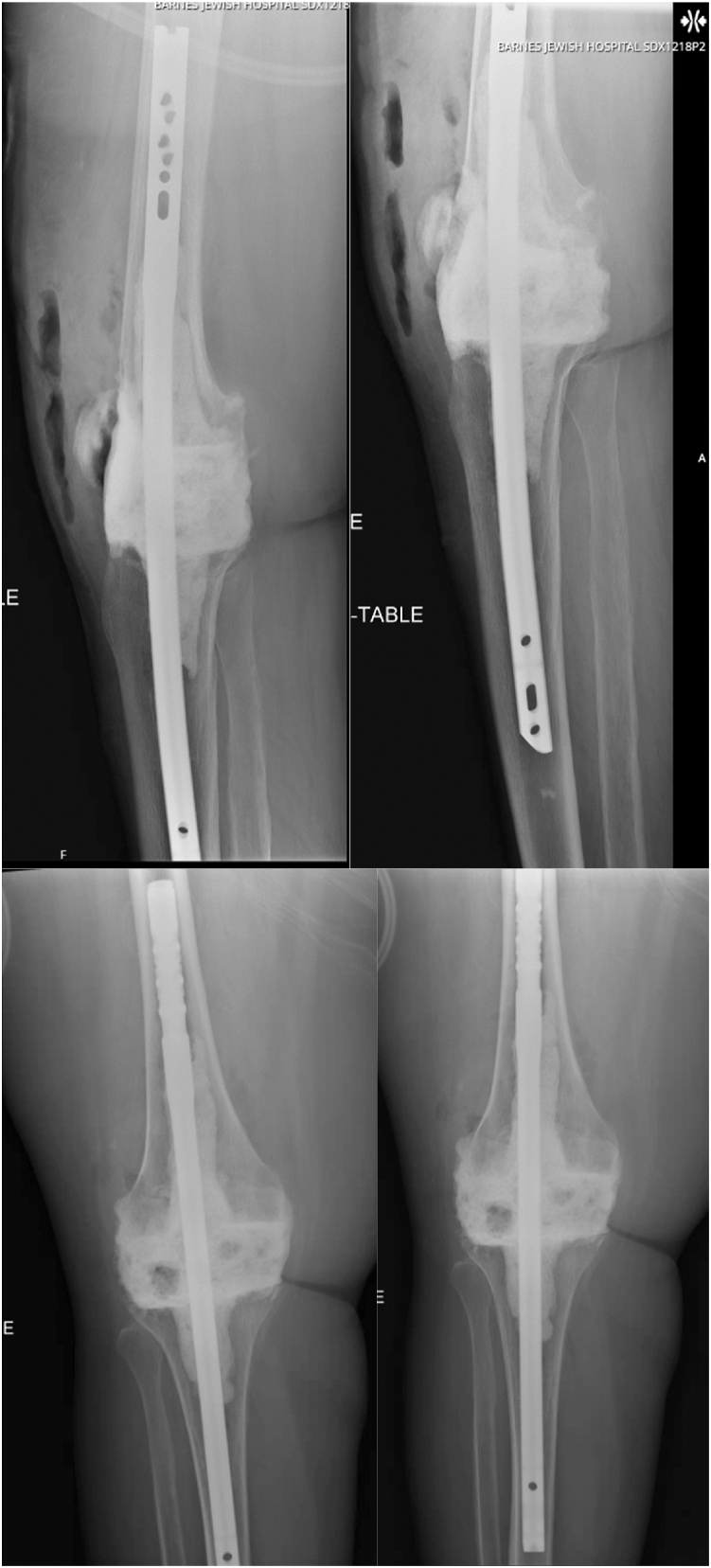
Standard anteroposterior and lateral radiographs capturing the proximal and distal extent of the nail are obtained postoperatively.

After the cement has hardened, a sterile Doppler should be used to check dorsalis pedis pulse following deflation of the tourniquet. Arthrotomy and skin closure may then be performed according to surgeon preference. Soft tissue defects should also be managed at this time. Standard anteroposterior and lateral radiographs should be obtained postoperatively and patient may be weight-bearing as tolerated on the operative extremity without a brace (Fig. 8).

### Spacer removal

Nail removal may be required for spacer conversion to revision TKA following successful eradication of infection, or for spacer exchange if the patient is unable to clear their infection. The patient should be again prepped and draped in standard fashion, positioned supine with nonsterile tourniquet high on the operative thigh. Previous midline approach to operative knee should be performed similarly to previously described. Osteotomes may then be used to remove cement from joint space, taking care not to plunge osteotome deep toward the back of the knee during removal. After all cement has been carefully removed from the joint space using a pituitary rongeur, a metal-cutting burr may be used to cut the femoral nail in half. The knee may then be flexed, the femoral and tibial metaphyseal cement can then be removed with osteotomes and/or cement splitters, and each half of the nail may then be removed from the femoral and tibial canal using a vice grip and mallet.

## Discussion

The femoral nail and cement static spacer construct is a reliable option for treating prosthetic knee joint infections. It is of special importance in the systemic immune-status type C group, for whom articulating spacers may be unrealistic. Patients can weight-bear as tolerated on this construct immediately without a brace. This is also a cost-effective technique, as many linked nail devices currently available to allow immediate full weight-bearing postoperatively are much more expensive. Reimplantation after infection eradication is safely achieved in at least 64% of patients [9]. Reimplantation is performed using revision total knee prostheses, commonly a varus/valgus constrained construct or a rotating hinge. When this technique is performed, implant removal is simple with minimal additional bone loss.

## Summary

The femoral nail and cement static spacer technique presented here may be used to provide a durable solution for the first stage of 2-stage revision arthroplasty for chronic periprosthetic knee joint infection, allowing for brace-free full weight-bearing immediately following the procedure. Insertion and removal are relatively simple and respect the surrounding soft tissue envelope, which facilitates potential replantation.

## Conflicts of interest

The authors declare there are no conflicts of interest.

For full disclosure statements refer to https://doi.org/10.1016/j.artd.2025.101630.

## CRediT authorship contribution statement

**Katherine Mistretta:** Writing – review & editing, Writing – original draft. **Caroline Granger:** Writing – review & editing. **Joseph Kromka:** Writing – review & editing. **Andrew M. Schneider:** Writing – review & editing, Conceptualization.

## Appendix A Supplementary data

Conflict of Interest Statement for Granger

Conflict of Interest Statement for Kromka

Conflict of Interest Statement for Mistretta

Conflict of Interest Statement for Schneider
